# Mexiletine rescues a mixed biophysical phenotype of the cardiac sodium channel arising from the SCN5A mutation, N406K, found in LQT3 patients

**DOI:** 10.1080/19336950.2018.1475794

**Published:** 2018-08-01

**Authors:** Rou-Mu Hu, David J. Tester, Ryan Li, Tianyu Sun, Blaise Z. Peterson, Michael J. Ackerman, Jonathan C. Makielski, Bi-Hua Tan

**Affiliations:** a Heart Center & Beijing Key Laboratory of Hypertension, Beijing Chaoyang Hospital, Capital Medical University, Beijing, China; b Division of Cardiovascular Medicine, Department of Medicine, University of Wisconsin, Madison, Wisconsin, USA; c Departments of Medicine, Pediatrics, and Molecular Pharmacology and Experimental Therapeutics, Mayo Clinic, Rochester, MN, USA; d Departments of Pediatrics, and Cellular & Molecular Physiology, Pennsylvania State University College of Medicine, Hershey, PA, USA

**Keywords:** Mexiletine, mixed phenotype, mutation, SCN5A, sodium channel

## Abstract

Introduction: Individual mutations in the *SCN5A*-encoding cardiac sodium channel α-subunit usually cause a single cardiac arrhythmia disorder, some cause mixed biophysical or clinical phenotypes. Here we report an infant, female patient harboring a N406K mutation in SCN5A with a marked and mixed biophysical phenotype and assess pathogenic mechanisms. Methods and Results: A patient suffered from recurrent seizures during sleep and torsades de pointes with a QTc of 530 ms. Mutational analysis identified a N406K mutation in SCN5A. The mutation was engineered by site-directed mutagenesis and heterologously expressed in HEK293 cells. After 48 hours incubation with and without mexiletine, macroscopic voltage-gated sodium current (*I*_Na_) was measured with standard whole-cell patch clamp techniques. SCN5A-N406K elicited both a significantly decreased peak *I*_Na_ and a significantly increased late *I*_Na_ compared to wide-type (WT) channels. Furthermore, mexiletine both restored the decreased peak *I*_Na_ of the mutant channel and inhibited the increased late *I*_Na_ of the mutant channel. Conclusion: SCN5A-N406K channel displays both “gain-of-function” in late *I*_Na_ and “loss-of-function” in peak *I*_Na_ density contributing to a mixed biophysical phenotype. Moreover, our finding may provide the first example that mexiletine exerts a dual rescue of both “gain-of-function” and “loss-of-function” of the mutant sodium channel.

## Introduction

The gene *SCN5A* on chromosome 3 encodes the α-subunit of voltage-gated cardiac sodium channel (hNa_v_1.5) that is responsible for generating a large peak inward Na current (*I*_Na_) [1]. *I*_Na_ underlies initiation and propagation of action potentials in working myocardium (atrial and ventricular cells) and special conduction tissue (Purkinje cells etc.) [2]. Mutations in SCN5A are responsible for a spectrum of hereditary arrhythmias [3]. Phenotypic variability arises from the effects of the mutations on the hNa_v_1.5 biophysical properties. These effects usually fall into two categories, gain or loss of channel function [4]. In “gain-of-function” mutations, an increase in the persistent late *I*_Na_ during the action potential plateau, rather than in peak *I*_Na_ is typically thought to be responsible for the type 3 long QT syndrome (LQT3) phenotype whereby repolarization is delayed and the QT interval is prolonged on the surface electrocardiogram (ECG) [5]. In contrast, “loss-of-function” mutations in SCN5A cause a decrease in peak *I*_Na_, leading to phenotypes including Brugada syndrome (BrS), cardiac conduction disturbance (CCD), congenital sick sinus syndrome (SSS), atrial standstill, AV block, sudden infant syndrome, and familial atrial fibrillation [3,6]. Some SCN5A mutations have also been found to yield mixed clinical and/or biophysical phenotypes [7,8]. These mutations can cause both an increase in late *I*_Na_ and a decrease in peak *I*_Na_, thereby exhibiting gain-of-function and loss-of-function characteristics simultaneously.

The SCN5A mutation N406K (asparagine replaced with lysine at position 406) was reported previously in two separate patients associated with LQT3 phenotype [9,10]. Both studies reported significantly prolonged QTc interval on surface ECG and increased late *I*_Na_ in functional studies. One of these two studies also showed this mutant exhibited a decreased peak *I*_Na_ as loss-of-function [9]. Whether the N406K mutant has a mixed biophysical phenotype or not is still unknown.

We describe here a patient also haboring this N406K mutant. Using patch clamp technique, we characterized the mutated channel in HEK 293 cells and identified both “gain-of-function” and “loss-of-function” effects in this mutation. Furthermore, we applied the antiarrhythmic drug mexiletine by incubation with sodium channels and discovered that mexiletine could both elevate the decreased peak *I*_Na_ and inhibit the increased late *I*_Na_ of the SCN5A-N406K channel, providing pharmacological rescue of defective mutated sodium channel.

## Methods

### Study subjects

The study conforms to the principles outlined in the Declaration of Helsinki and was approved by the Research Ethics Committee of the Mayo Foundation Institution Review Board. Written informed consent was obtained from all participants.

### Mutation screening

Genomic DNA was extracted from peripheral blood lymphocytes as previously described and screened for the entire open-reading frames of 15 LQTS-susceptibility genes by PCR, denaturing high-performance liquid chromatography (HPLC) and direct DNA sequencing.

### Plasmid construction

The N406K mutation was generated using QuickChange site-directed mutagenesis kits according to the manufacturer’s instructions (Stratagene, La Jolla, CA) and was engineered into the common splice variant of human cardiac voltage-dependent Na channel SCN5A/hNav1.5 [containing a glutamine at position 1077, we note as Q1077 (Genbank accession no. AC1377587)] in the pcDNA3 plasmid vector (Invitrogen, Carlsbad, CA) as previously reported [11,12]. All clones were sequenced to confirm integrity and to ensure the presence of the target mutations without other substitutions.

### Heterologous expression and drug treatment

The WT- or N406K-SCN5A cDNA was transiently co-transfected with pMaxGFP cDNA, at a ratio of 5:1, into HEK293 cells with Superfect (Qiagen, Valencia, CA) following manufacturer’s recommended protocol [13,14]. After 3–5 hours of incubation, the transfection reagent-DNA mixture was replaced with 3 ml of normal culture medium with or without 500 μM mexiletine, and the cells were incubated at 37°C for 48 hours. Before the electrophysiological recording, the plates containing the cells were removed from the 37°C incubator, growth medium was aspirated off the plates with or without drug, and the cells were treated with a 0.25% trypsin-1 mM EDTA solution (GIBCO-BRL) and transferred to a fresh tube along with 2 ml of normal culture medium and directly to the experimental chamber for electrophysiological recording [15].

### Standard electrophysiological measurements

Macroscopic voltage-gated *I*_Na_ was measured 24 hours after transfection with the standard whole-cell patch clamp technique at a temperature of 22–24°C in HEK 293 cells expressed the GFP (“green cells”). Cells were continuously perfused with bath (extracellular) solution containing 140 mM NaCl, 4 mM KCl, 1.8 mM CaCl_2_, 0.75 mM MgCl2 and 5 mM HEPES (pH 7.4 set with NaOH). The pipette (intracellular) solution contained 120 mM CsF, 20 mM CsCl_2_, 5 mM EGTA and 5 mM HEPES and was adjusted to pH 7.4 with CsOH. Microelectrodes were made of borosilicate glass with a puller (P-87, Sutter Instrument Co, Novato, CA, USA) and were heat-polished using a microforge (MF-83, Narishige, Tokyo, Japan). The resistances of microelectrodes ranged from 1.0 to 2.0 MΩ when filled with recording solution. Voltage clamp was generated by Axopatch 200B amplifier (Axon Instruments, Foster City, CA) and controlled using pClamp software 9.0. The series-resistance was compensated. Membrane current data were digitized at 100 kHz, low-pass filtered at 5 kHz, and then normalized to membrane capacitance. The standard voltage clamp protocols are presented with the data and have been previously described [16].

### Flow cytometry and Quantification of Cell Surface Expression

HEK293 cells were transiently co-transfected with HA-tagged SCN5A-WT or HA-tagged SCN5A-N406K respectively. After 48 hours of transfection and with or without mexiletine treatment, the transfected HEK293 cells were harvested by incubation with 0.5 mM EDTA-PBS for 10 min at 37°C and washed with RPMI 1640 supplemented with 1 mM EDTA (pH 7.4), 3%FCS, and 0.02% azide (staining medium). The FITC-conjugated anti-HA antibody (Sigma-Aldrich) incubations were performed in staining medium at 4°C and then washed by PBS supplemented with 1 mM EDTA (pH 7.4) and 1% FCS. The stained cells were examined for surface expression with FACSCalibur (BD Biosciences, San Jose, CA).

### Statistical analysis

All data points are shown as the mean value and SE. Determinations of statistical significance were performed using a Student’s t-test for comparisons of two means or using one-way ANOVA for comparisons of multiple means. A P value of < 0.05 was considered statistically significant.

## Results

### Clinical characterization

**Figure 1.**
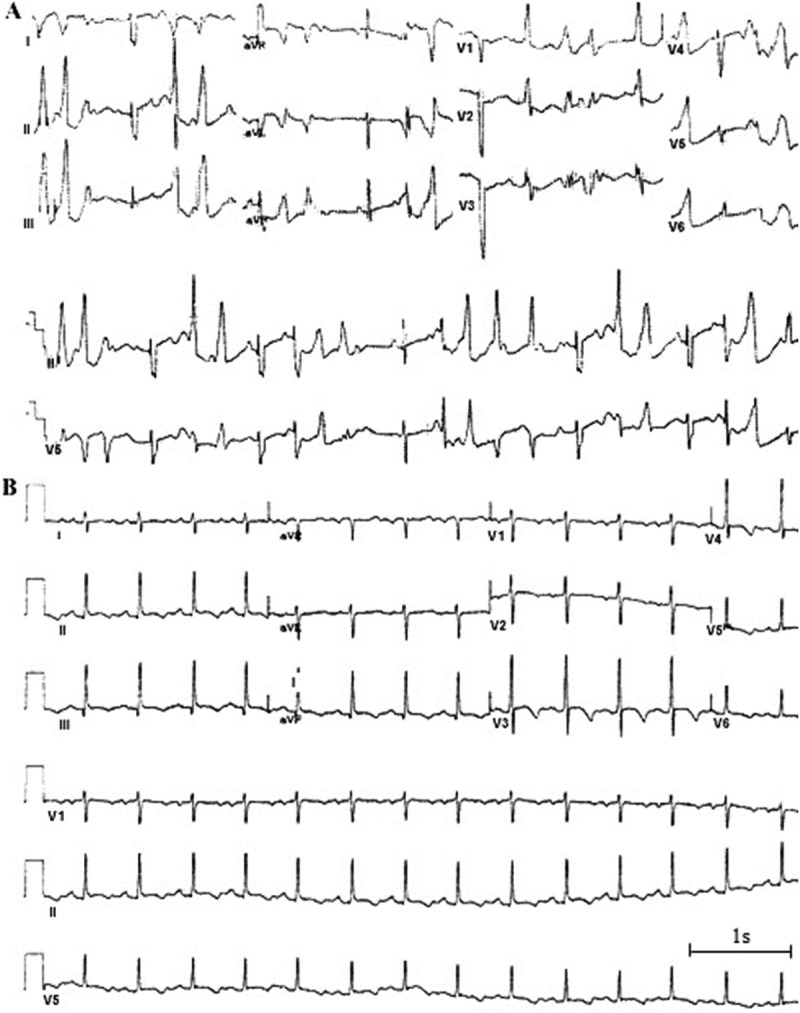
ECG phenotype (A) ECG of the patient at 1 day showed the infant had polymorphic ventricular tachycardia (B) ECG of the patient at 3 months showed a prolonged QT interval (QTc = 530 ms).

The patient was found to have polymorphic ventricular tachycardia (Figure 1) and many episodes of torsades de pointes (TdP) at birth. She had a history of multiple seizures beginning at age 3 months. The QT interval was significantly prolonged (QTc = 530ms) according to surface ECG (Figure 1(b)). None of her family members had similar symptoms.

### Genetic analysis

**Figure 2.**
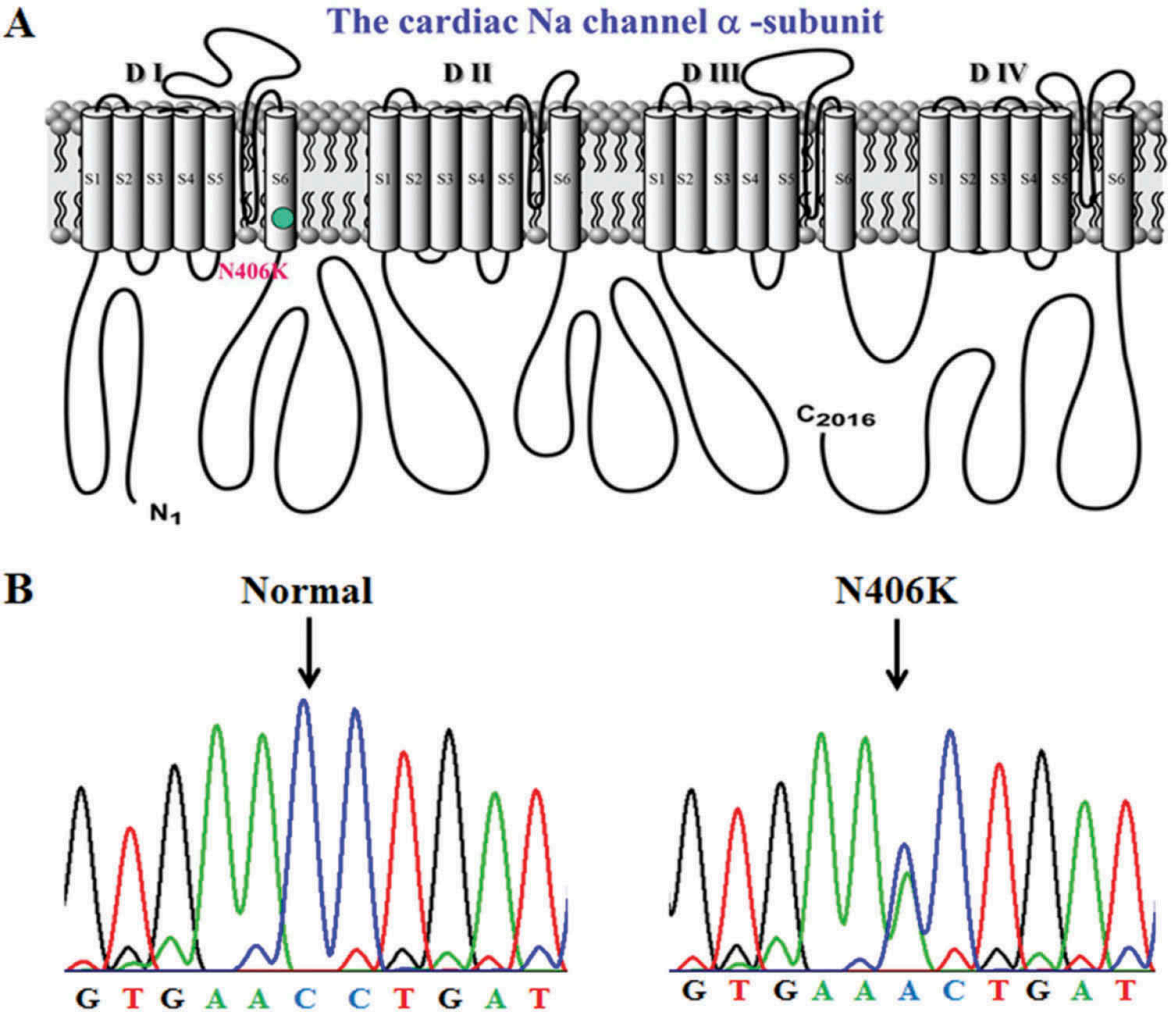
(A) Topological diagram of the voltage-dependent sodium channel α-subunit hNav1.5 showing a missense mutation, N406K on the linear topology of the S6 segment at domain I of cardiac Na channel. (B) Sequence chromatogram showing normal (left panel) and a missense mutation in codon 406 of SCN5A resulting in a replacement of an asparagine by a lysine (N406K).

Fifteen LQTS candidate genes were screened in the patient. Comprehensive open-reading frame/splice site mutational analysis for the *SCN5A* gene revealed a missense mutation (N406K). N406K (c. 1218C > A) was a single-base transition at nucleotide 1218 in exon 10 that resulted in the substitution of asparagine in codon 406 to lysine in S6 segment of hNav1.5 domain I (Figure 2). N406K was absent from 1300 SCN5A alleles [17] of an ethnically matched, healthy control cohort.

### Current expression and gating properties of SCN5A-N406K

**Figure 3.**
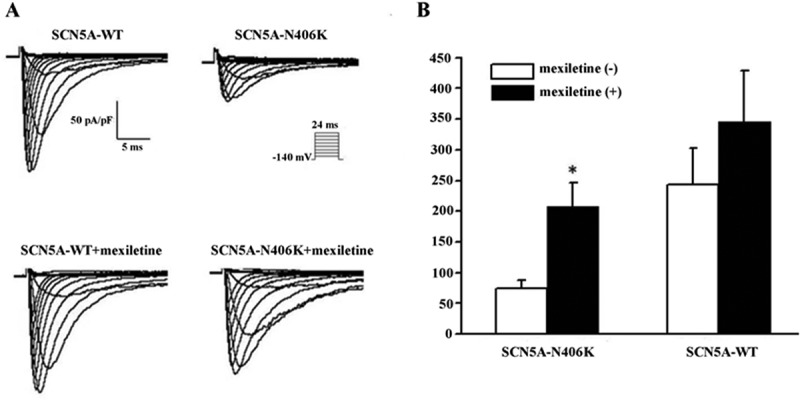
Electrophysiological properties of SCN5A-WT and SCN5A-N406K in HEK293 cells incubation w/and w/o mexiletine. (A) Whole-cell current traces from representative experiments of each group. (B) Summary data of peak *I*_Na_ density of each group. Currents were elicited by test depolarization to 24ms from a holding potential of −140 mV. Symbols represent means and bars represent SEM. *, P < 0.01 versus SCN5A-WT w/o mexiletine.

HEK-293 cells transiently expressing the SCN5A-WT or SCN5A-N406K mutant were voltage clamped after 24 hours of transfection. Mean peak *I*_Na_ densities for WT and mutant channels were compared for experiments performed on the same day in order to reduce variability. Examples of macroscopic *I*_Na_ traces for WT and mutant channels are shown in Figure 3 and summary data are given in Table 1. SCN5A-N406K mutant channels displayed a clear and signficant reduction of ~ 60%-67% in peak *I*_Na_ compared to SCN5A-WT (Figure 3(a,b), n = 18–22, p < 0.01, and Table 1).

**Table 1.** Voltage-dependent gating parameters of each groups and mexiletine rescue of SCN5A-N406K in heterologous expression system.

	SCN5A-WT		SCN5A-N406K	
Peak *I*_Na_ (pA/pF)		n		n
\MEX(-)	−243 ± 60	18	−75 ± 13*	22
MEX(+)	−345 ± 84	12	−208 ± 39	13
Activation V_1/2_ (mV)				
MEX(-)	−40 ± 5.3	10	−43 ± 1.0	16
MEX(+)	−41 ± 1.2	9	−42 ± 1.2	8
Inactivation V_1/2_ (mV)				
MEX(-)	−83 ± 4.0	8	−80 ± 1.8	9
MEX(+)	−81 ± 4.0	7	−83 ± 4.0	8
Recovery				
τ_f_ (ms)	1.8 ± 0.3	7	1.2 ± 0.1	10
τ_s_ (ms)	33 ± 7.4	7	34 ± 5.5	10
A*s* (%)	21 ± 1.0	7	24 ± 2.8	10

* P < 0.01 v.s.WT

The fitted values of voltage-dependent gating parameters represent the mean SEM for number of experiments in the parentheses. These parameters were obtained from fitting the individual experiments to the appropriate model equations. For the Boltzmanm fits the parameters of V1/2 are the midpoint of activation and inactivation, and K is the slope factor.

**Figure 4.**
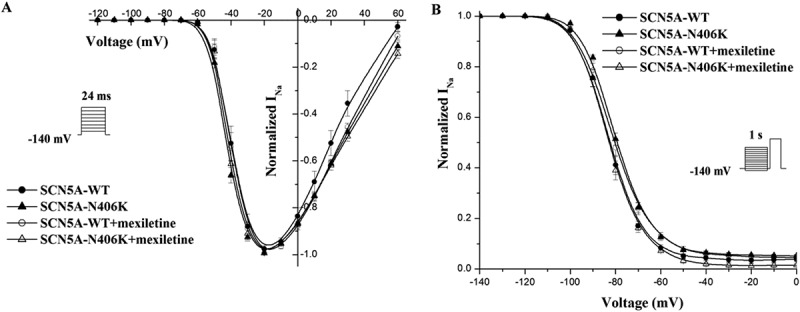
Voltage-dependence of steady-state activation and inactivation was measured from HEK293 cells transiently expressing WT or mutant SCN5A incubation w/and w/o mexiletine. (A) SCN5A-N406K did not alter steady-state activation parameters significantly compared to SCN5A-WT. (B) SCN5A-N406K did not affect steady-state inactivation parameters significantly compared with SCN5A-WT.

To investigate the gating properties of mutant Na channels, we analyzed the kinetic parameters concerning activation and inactivation of SCN5A-N406K of SCN5A-N406K and compared the data with SCN5A-WT. Peak *I*_Na_ at each voltage were normalized to the whole cell capacitance and plotted against test voltages (Figure 4 (a,b)). Mutant channels and WT channels showed no difference in steady-state activation midpoints, steady-state inactivation midpoints or recovery parameters (Table 1).

### Late I_Na_ of SCN5A-N406K mutant channels

**Figure 5.**
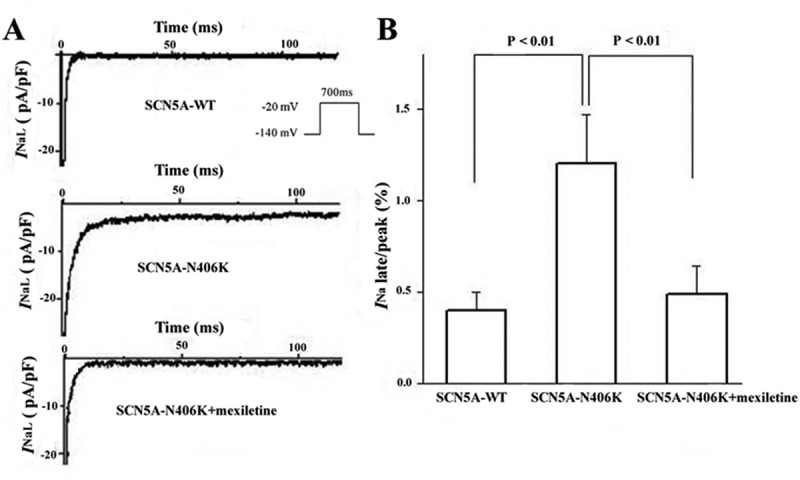
Late *I*_Na_ of SCN5A-WT and SCN5A-N406K in HEK293 cells incubation w/and w/o mexiletine. (A) Representative traces showing increased late *I*_Na_ associated with SCN5A-N406K compared with SCN5A-WT and the increased late *I*_Na_ could be inhibited by mexiletine. (B) Summary data for late *I*_Na_ normalized to peak *I*_Na_ after leak subtraction. Currents were elicited by a test depolarization pulse from −140 mV to −20mV for 700ms after the initiation of the depolarization. Symbols represent means and bars represent SEM.

Late *I*_Na_ for both the N406K mutant and WT channels were measured as the leak subtracted inward current remaining at the end of a 700-ms-long depolarization and expressed as a ratio of late/peak *I*_Na_ as previously reported [18,19]. Representative late *I*_Na_ traces are shown in Figure 5. Compared to WT channels, N406K mutant channels elicited significantly increased late *I*_Na_ by 3 fold (Figure 5 (a,b), n = 7, p < 0.01).

### SCN5A-N406K caused slower decay of I_Na_

**Figure 6.**
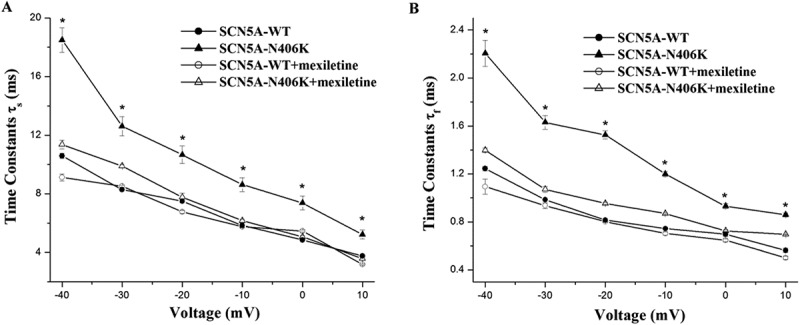
Decay of macroscopic current. (A) Voltage dependence of inactivation slow time constants. When compared with SCN5A-WT, SCN5A-N406K showed larger slow component (Τ_s_) values through a wide range of test potentials from −40 mV to 10 mV. (B) Voltage dependence of inactivation fast time constants. When compared with SCN5A-WT, SCN5A-N406K showed larger fast component (Τ_f_) values through a wide range of test potentials from −40 mV to 10 mV. Both effects can be abolished by mexiletine. *P < 0.05 vs. SCN5A-WT.

Time constants (Τ_s_, Τ_f_) were obtained from 2-exponential fits of decay phase of macroscopic *I*_Na_ measured at various potentials. When compared with SCN5A-WT channel, the SCN5A-N406K channel significantly increased and values through a wide range of test potentials (P < 0.05; Figure 6 (a,b)). This suggested that inactivation of *I*_Na_ in SCN5A-N406K was impaired.

### Reduced Cell Surface Expression of N406K

**Figure 7.**
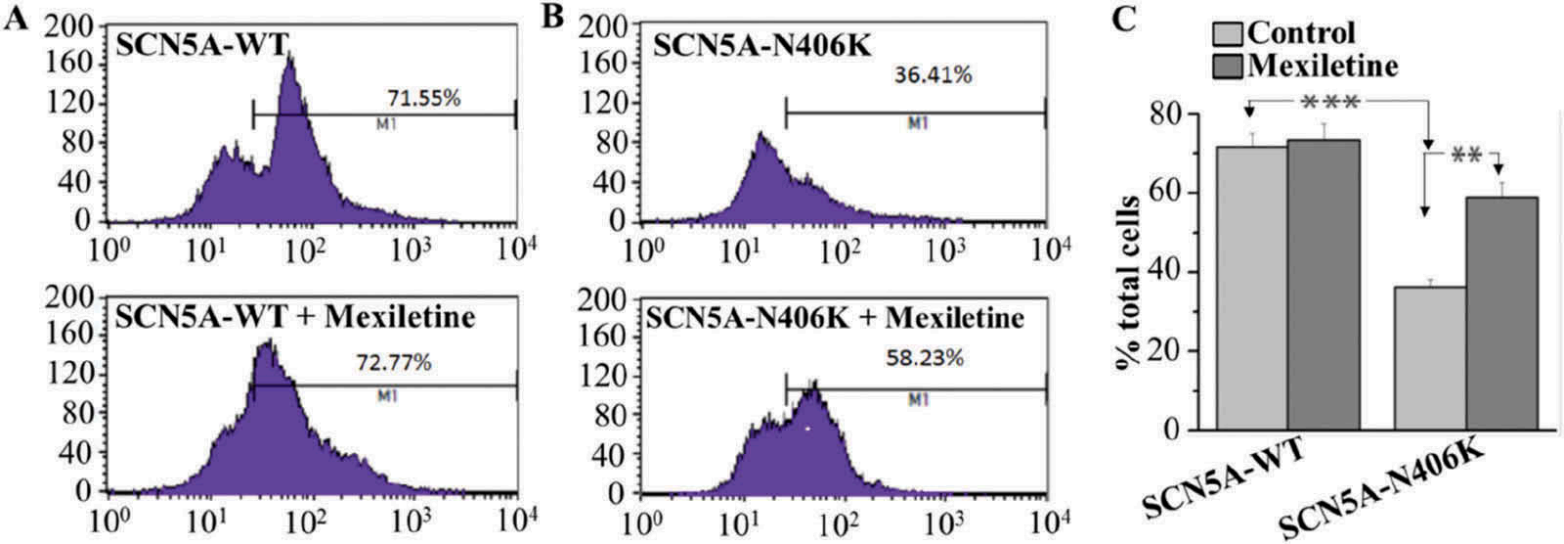
Flowcytometric analysis of cell surface expression of the HA-tagged SCN5A WT and mutant channels. HEK293 cells transiently co-transfected with HA-tagged SCN5A-WT, HA-tagged SCN5A-N406K respectively. After 48 hours of transfection and with or without mexiletine treatment, the cells were harvested and stained by FITC-conjugated anti-HA antibody for quantitative the plasma membrane expression of HA-tagged channels by flow cytometry. (A-B): the cell count curve of SCN5A-WT (A) and SCN5A-N406K (B) in the absence of mexiletine (upper panel) and presence of mexiletine (Lower panel). Marker 1 (M1) was established at a point containing 5% of negative control. (C) the summary data to show the percentage of counted cells which the plasma membrane expressed HA-tagged SCN5A-WT and HA-tagged SCN5A-N406K. ***P < 0.001, compared with WT; ** P < 0.01, compared to the non-treatment control.

The N406K mutation has been shown to decrease peak *I*_Na_ density. One explanation for this result is that N406K might not be processed correctly, as has been reported for a number of “loss-of-function” mutations. To test this idea, an extracellular HA epitope was introduced into both WT and N406K channels and their cellular localization was studied. We used flowcytometry to discover that N406K reduced SCN5A cell surface expression compared with WT. The N406K mutation was noted to decrease cell surface expression of SCN5A compared with SCN5A-WT (Figure 7).

### Effects of mexiletine on SCN5A-N406K mutant channels

HEK-293 cells expressing WT or N406K mutant channels were incubated for 48 hours with 500 μM mexiletine at physiological temperature. Currents were measured after washout of mexiletine, which was present only during the incubation period. Mexiletine treatment increased current density in both WT and mutant channels, but more markedly for N406K (Figure 3). After 48 h incubation with mexiletine, peak *I*_Na_ for WT channels were increased by 1.4-fold compared to non-incubation, however showing no significant difference. In contrast, SCN5A-N406K mutant channels incubated with mexiletine exhibited significantly increased peak *I*_Na_ by 2.8-fold compared with control non-incubated mutant channels (p < 0.01) (Figure 4). Interestingly, Late *I*_Na_ for SCN5A-N406K mutant channels incubated with mexiletine were markedly decreased by ~ 58%-60% compared to mutant channels without mexiletine incubation (p < 0.01) (Figure 4). Notably, the time constants (Τ_s_, Τ_f_) of current decay of SCN5A-N406K mutant channels after incubation with mexiletine also returned to normal levels (Figure 6 (a,b)). The decrease in cell surface expression caused by SCN5A-N406K mutants was reversed by mexiletine (Figure 7), further supporting the hypothesis that mexiletine could increase the peak *I*_Na_ of SCN5A N406K mutation through rescuing the trafficking defect of SCN5A-N406K.

## Discussion

### Major findings

In the present study, we identified the SCN5A-N406K mutation in a female patient presented with frequent episodes of TdP and marked QT interval prolongation consistent with LQT3 phenotype. Functional assays using a heterologous expression system revealed that SCN5A-N406K exhibited a “gain-of-function” effect (i.e. an increase in late *I*_Na_) as well as a “loss-of-function” effect (i.e. a decrease in peak *I*_Na_) which is consistent with the previous report by Kato et al [9]. Furthermore, both the “gain-of-function” effect and the “loss-of-function” effect could be reversed by mexiletine, a Class I antiarrhythmic drug.

Spencer et al [10] discovered both action potentials and calcium transients were markerly prolonged in patient-derived induced pluripotent stem cells expressing SCN5A-N406K. The enhanced calcium transients were thought to account for the polymorphic ventricular tachycardia the patient has at birth.

### Mixed biophysical phenotype of SCN5A-N406K

The mutant channel SCN5A-N406K showed a gain of function biophysical phenotype resulting in seizures during sleep and TdP of the patient. The dramatic increased late *I*_Na_ found for SCN5A-N406K is typical of LQT3 [20]. The persistent Na current during action potential plateau is due to the failure of fast Na channel inactivation [8]. The remarkably slowed decay of currents observed in SCN5A-N406K mutant channels also indicated that the inactivation was impaired. Decelerated decay of macroscopic currents, stemming from delayed onset of inactivation, often occurs in combination with persistent Na current [21]. This destabilized inactivation shifts the ionic balance toward the inward current and delays repolarization, thus the action potential duration and the corresponding QT interval are prolonged consequently [22]. Unlike LQT1 and LQT2, clinical events in LQT3 tend to occur during sleep or at rest, so that LQT3 is more insidious and more lethal.

Moreover, SCN5A-N406K also exhibited decreased peak *I*_Na_ when expressed in HEK 293 cells, recognized as a “loss-of-function” biophysical phenotype. Such “loss-of-function” is the characteristic of Brugada syndrome (BrS) or cardiac conduction disturbance (CCD) [4]. However, there was no ECG evidence of Brugada-type ST elevation, right bundle branch block morphology or conduction disorders seen in this patient. Reduced sodium current is thought to exaggerate discrepancy in the action potential duration between the inner and outer layers of ventricular muscle, thereby introducing increased electrophysiological heterogeneity [23]. There are three common mechanisms that mutations in the SCN5A can lead to loss of function [8,15,24]: (1) incomplete transcription due to nonsense mutations that cause the channel protein not to be produced. (2) Channel proteins are produced but intracellular trafficking of the channel proteins is impaired thus membrane surface expression of the channel protein are decreased. (3) Channel proteins are produced and traffic to the surface are dysfunctional because of altered channel-gating properties, including disrupted activation, enhanced inactivation as well as impaired recovery from inactivation. In our study, N406K is a missense not nonsense mutation; mutant channels showed no difference in steady-state activation, steady-state inactivation or recovery parameters compared with WT channels. Therefore we speculate that the most probable mechanism for loss-of-function in N406K is impared trafficking of the channel proteins.

SCN5A mutations with characteristics of both phenotypes have been reported [7]. It seems paradoxical that a mutation could cause both “gain-of-function” and “loss-of-function” of sodium channel. This apparent pardox is resolved when considering the different timing of sodium current effects: “gain-of-function” usually refers to increased late *I*_Na_ during repolarization and “loss-of-function” generally refers to decreased peak or early *I*_Na_ during depolarization. The most common mixed LQT3/BrS mutation in SCN5A is probably E1784K [25]. Patients with this mutant can present with either or both prolonged QT intervals and ST segment abnormalities in an ECG. In contrary to N406K, E1784K does not alter channel expression, but rather alters channel gating (shifts the midpoint of the channel conductance-voltage relationship to more depolarized membrane potentials and accelerates the rate of channel fast inactivation) to increase late *I*_Na_ and decrease peak *I*_Na_ .The N406K mutation reported here provides another example of a mixed biophysical phenotype with “gain-of-function” for late *I*_Na_ and “loss-of-function” for peak *I*_Na_.

### Mexiletine: Dual effect on mixed biophysical phenotype of SCN5A-N406K

As “gain-of-function” SCN5A mutations induce an excess of sodium entering into the cells, sodium channel blockade represents a rational approach for gene-specific therapy in LQT3. Preliminary experimental and clinical evidences claim that mexiletine, a sodium channel blocker targeted in the inactivated state [26], could preferentially reduce the elevated late *I*_Na_ [27] and shorten the APD in a cellular model of LQT3 [28,29] and the QT interval in LQT3 patients [30,31]. Liu et al reported that mexiletine produces more significant QRS widening when APD prolongation is induced by late *I*_Na_ enhancer than *I*_Kr_ blocker in the rabbit ventricular wedges, which indicates that the enhanced late *I*_Na_ may be the consequence due to the failure of fast Na channel inactivation [32]. We have also validated the rescuing effect that mexiletine could inhibit the increased late *I*_Na_ of SCN5A-N406K. Interestingly, both the fast time constant (Τ_f_) and slow time constant (Τ_s_) values of SCN5A-N406K incubated with mexiletine were much smaller than the mutant channel incubated without mexiletine. This implied that mexiletine might block the increased late *I*_Na_ by correcting the imparied inactivation of the mutant channel.

In terms of “loss-of-function” SCN5A mutation, mexiletine was able to restore decreased peak *I*_Na_ by rescuing the proper localization of the protein [33]. In our study, the SCN5A-N406K showed a distinct reduced peak *I*_Na_ that was successfully rescued by mexiletine. Valdivia et al reported M1766L as the first SCN5A mutation expression defect rescued by mexiletine [34]. The M1766L mutation caused a significant decrease in peak *I*_Na_ density due to reduced sodium channel expression. Incubation with mexiletine partially rescued the defective expression. M1766L also showed a drastic increase in late *I*_Na_, however, mexiletine did not inhibit late *I*_Na_ in this mutation. Moreau et al. showed two SCN5A mutations (A124D and V1378M) caused dramatically reduced peak *I*_Na_ density without changes in the kinetics and gating properties of the mutant channel [35]. Mexiletine could partially restore the current density of V1378M but had no effect on the current density of A124D. Incubation at 25°C and drugs targeting ER resident proteins could partially rescue the current density of these two mutations. This suggests that the effectiveness of mexiletine to rescue trafficking defects may depend on the location of the mutation.

It should be noted that the doses of mexiletine used in this study for rescue in the heterologous system are greater than therapeutic levels. Mexiletine rescue, however, could be considered a “proof of principle”, and maybe other rescue drugs could be found to rescue in concentrations nontoxic to patients.

In regard to mixed clinical phenotype/overlap syndrome, therapeutic strategies are more complex. The most frequently reported overlap of SCN5A channelopathies is the concomitant occurrence of BrS and CCD. Pharmacological treatment of BrS/CCD overlap seems less challenging, as both phenotypes are caused by the same mutation-specific loss-of-function mechanism. On the contrary, the concomitant presentation of LQT3 plus either BrS, CCD or even both may create a much more complicated situation [36], because a potentially efficient therapy should address both the inhibition of a gain-of-function effect and the rescue of a loss-of-function effect in the same mutant channel. We discovered mexiletine could elevate the reduced peak sodium current and inhibit the increased late sodium current at the same time, and partially restore the function of SCN5A-N406K mutant channel with a mixed biophysical phenotype. Whether or not mexiletine have similar rescuing effect on mixed clinical phenotype represents a possible future direction for this work.

It is important to emphasize that our study in a “minimalist” heterologous system may not reflect what occurs in the cardiomyocytes. The SCN5A complex in native tissue has many more components such as β subunits which we did not co-express in the heterologous cell model, and some interacting proteins found in native heart could have modulatory roles. Also these clones do not contain the introns or promoters that might affect expression in the cardiomyocytes. Our experiments in the heterologous system only suggest a possible biophysical phenotype that may lead to the clinical syndromes, but further studies in a more native environment of cardiomyocytes is necessary to describe the full pathogenetic pathway.

In conclusion, we describe the SCN5A-N406K mutation that exhibits both “gain-of-function” in late *I*_Na_ as other LQT3 mutations, and “loss-of-function” in peak *I*_Na_ density. Furthermore, the antiarrhythmic drug mexiletine could inhibit the increased late *I*_Na_ in SCN5A-N406K and restore the decreased peak *I*_Na_ density as well, suggesting a dual rescuing effect on mixed biophysical phenotype.
